# Protocol for the evaluation of the population-level impact of Zimbabwe’s prevention of mother-to-child HIV transmission program option B+: a community based serial cross-sectional study

**DOI:** 10.1186/s12884-018-2146-x

**Published:** 2019-01-08

**Authors:** Aybuke Koyuncu, Mi-Suk Kang Dufour, Sandra Irene McCoy, Sergio Bautista-Arredondo, Raluca Buzdugan, Constancia Watadzaushe, Jeffrey Dirawo, Angela Mushavi, Agnes Mahomva, Frances Cowan, Nancy Padian

**Affiliations:** 1 University of California Berkeley, Berkeley, USA; 2 Division of Prevention Science, University of California San Francisco, San Francisco, USA; 3 Consorcio de Investigación Sobre VIH/SIDA/TB, Cuernavaca, Mexico; 4 Centre for Sexual Health and HIV Research Zimbabwe, Harare, Zimbabwe; 5 Ministry of Health and Child Care, Harare, Zimbabwe; 6 Elizabeth Glaser Pediatric AIDS Foundation, Harare, Zimbabwe; 7 Liverpool School of Tropical Medicine, Liverpool, UK

**Keywords:** Impact evaluation, Mother-to-child transmission of HIV (MTCT), Prevention of mother-to-child HIV transmission (PMTCT), Antiretroviral therapy (ART)

## Abstract

**Background:**

WHO recommends that HIV infected women receive antiretroviral therapy (ART) minimally during pregnancy and breastfeeding (“Option B”), or ideally throughout their lives regardless of clinical stage (“Option B+”) (Coovadia et al., Lancet 379:221–228, 2012). Although these recommendations were based on clinical trials demonstrating the efficacy of ART during pregnancy and breastfeeding, the population-level effectiveness of Option B+ is unknown, as are retention on ART beyond the immediate post-partum period, and the relative impact and cost-effectiveness of Option B+ compared to Option A (Centers for Disease Control and Prevention, Morb Mortal Wkly Rep 62:148–151, 2013; Ahmed et al., Curr Opin HIV AIDS 8:473–488, 2013). To address these issues, we conducted an impact evaluation of Zimbabwe’s prevention of mother to child transmission programme conducted between 2011 and 2018 using serial, community-based cross-sectional serosurveys, which spanned changes in WHO recommendations. Here we describe the rationale for the design and analysis.

**Methods/design:**

Our method is to survey mother-infant pairs residing in the catchment areas of 157 health facilities randomly selected from 5 of 10 provinces in Zimbabwe. We collect questionnaires, blood samples from mothers and babies for HIV antibody and viral load testing, and verbal autopsies for deceased mothers/babies. Using this approach, we collected data from two previous time points: 2012 (pre-Option A standard of care), 2014 (post-Option A / pre-Option B+) and will collect a third round of data in 2017–18 (post Option B+ implementation) to monitor population-level trends in mother-to-child transmission of HIV (MTCT) and HIV-free infant survival. In addition, we will collect detailed information on facility level factors that may influence service delivery and costs.

**Discussion:**

Although the efficacy of antiretroviral therapy (ART) during pregnancy and breastfeeding for prevention of mother-to-child transmission of HIV (PMTCT) has been well-documented in randomized trials, little evidence exists on the population-level impact and cost-effectiveness of Option B+ or the influence of the facility on implementation (Siegfried et al., Cochrane Libr 7:CD003510, 2017). This study will provide essential data on these gaps and will provide estimates on retention in care among Option B+ clients after the breastfeeding period.

**Trial registration:**

NCT03388398 Retrospectively registered January 3, 2018.

## Background

Despite the implementation of increasingly efficacious drug regimens for the prevention of mother-to-child HIV transmission (PMTCT), in 2016,160,000 children became infected with HIV worldwide, of whom 48% lived in eastern and southern Africa [1]. Most pediatric HIV infections are acquired through mother-to-child transmission (MTCT), which can occur during pregnancy, delivery or breastfeeding [2]. In the absence of antiretroviral prophylaxis (ARV) or antiretroviral therapy (ART), MTCT ranges between 15 and 45% [3]. While the use and efficacy of ART during pregnancy and breastfeeding for PMTCT under controlled circumstances has been well documented [4, 5], the population-level effectiveness in real-world conditions has not [6].

Following the 2010 revision of the WHO PMTCT guidelines for resource-poor settings [7, 8] and the 2011 UNAIDS plan to eliminate MTCT globally [6], scale-up efforts for PMTCT interventions were renewed and highly efficacious ARV prophylaxis regimens became available in many low-income countries (e.g., Option A). Compared to earlier guidelines, Option A of the 2010 WHO guidelines recommended lifelong ART for a larger group of HIV-infected women (i.e., CD4 ≤ 350 or clinical stage 3–4 vs. CD4 ≤ 200), and that ARV prophylaxis be provided earlier in the pregnancy (i.e., starting at 14 weeks instead of 28) [9]. The commitment to scale up PMTCT interventions was further renewed in 2013 when the WHO released updated guidelines recommending that all pregnant women receive antiretroviral therapy throughout their lives regardless of clinical stage (Option B+) [10, 11]. However, MTCT rates in low resource settings remain higher than the levels achieved in efficacy studies [12, 13] or better-resourced settings, reflecting the reality that PMTCT effectiveness is a function of *both* ART efficacy *and* the proportion of HIV-infected pregnant women engaged and retained in PMTCT services [14]. In addition to accelerating the scale-up of PMTCT services, UNAIDS and WHO have urged countries to conduct population-level impact evaluations of their PMTCT programs [6, 15] to assess the extent to which these programs avert pediatric infections and to monitor progress towards PMTCT elimination goals [14, 15]. Despite the current global call for PMTCT effectiveness studies in developing countries, few countries have so far assessed the population-level impact of their PMTCT interventions [16, 17]. Equally unknown is the cost-effectiveness of PMTCT interventions such as Option B+, which remains a critical pending question in the context of limited resources for HIV programming.

In Zimbabwe, eliminating MTCT poses a formidable public health challenge. An estimated 1.3 million people are living with HIV, with an estimated 4900 new HIV infections in children each year [18]. In 2011, Zimbabwe’s Ministry of Health and Child Care (MOHCC), intensified the existing PMTCT program and scaled up a nationwide program to accelerate the elimination of pediatric HIV/AIDS. Reaching 60 of 62 districts and approximately 1560 health facilities, MoHCC supported the implementation of Option A of the 2010 WHO guidelines, distributed point-of-care CD4 testing machines for determination of ART eligibility, and facilitated community mobilization to increase entry and retention in the PMTCT cascade. In November 2013, the MOHCC updated its guidance to Option B+ as recommended in the 2013 WHO guidelines.

The scale-up of the accelerated PMTCT program in Zimbabwe provides an opportunity to ascertain the effectiveness of PMTCT programs at a national level. We will combine three rounds of serial community-based cross-sectional serosurveys to evaluate the population-level impact of Option B+ in Zimbabwe. Our 2017–2018 endline serosurvey (post Option B+) will be combined with data from our previous serosurveys conducted in 2012 (pre-Option A standard of care) and 2014 (post-Option A/pre-Option B+). These three surveys will allow us to monitor population-level trends in MTCT and HIV-free infant survival over the previous 6 years and to assess the impact of Option B+ compared to i) pre Option A and ii) post Option A pre Option B+. We use a population-based approach, as estimates from facility-based studies by definition will be less likely to include women who do not attend clinics for antenatal care and delivery. As with many settings in sub-Saharan Africa, mobility and migration are also a concern in assessing outcomes as many women may move during the postpartum period and become lost or be considered lost to care from a facility when they change care locations. Our study design allows us to assess HIV-free infant survival and MTCT at a community level at 9–18 months postpartum, thus taking into account HIV transmissions occurring during pregnancy, labor and delivery and breastfeeding among both women who access PMTCT services and those that do not [17]. We will specifically collect information on antenatal care attendance and facility based delivery as well as changes in care location and gaps in care related to mobility/migration.

In addition to evaluating the impact of Option B+, we will collect facility level data to explore impact heterogeneity and cost-effectiveness by facility and community level factors (e.g. MTCT) allowing us to assess Option B+ cost-effectiveness compared to Option A. Finally, our endline survey will include a population-based assessment of retention in HIV care long enough for some mothers who received Option B+ to have weaned their infant thus permitting an evaluation of care beyond the post-partum period. Here we describe our methodological approach for this impact evaluation.

## Methods/design

### Specific objectives

The specific objectives of this study include:

- Objective 1a, 1b: Compare the population-level impact of Option B+ on HIV-free survival and MTCT among infants 9–18 months of age in Zimbabwe to: a) standard of care before Option A; and b) Option A.
- Objective 2: Use facility and community level data to assess heterogeneity of the impact of Option B+ on HIV-free survival and MTCT among infants 9–18 months of age by the extent of integration of PMTCT and ART services at health facilities, among other factors.
- Objective 3: Assess retention of mothers in ART services 19–36 months postpartum.
- Objective 4a, 4b: Determine the cost-effectiveness of Option B+ compared to: a) the standard of care before Option A; and b) Option A.

### Study site and population

**Fig. 1:**
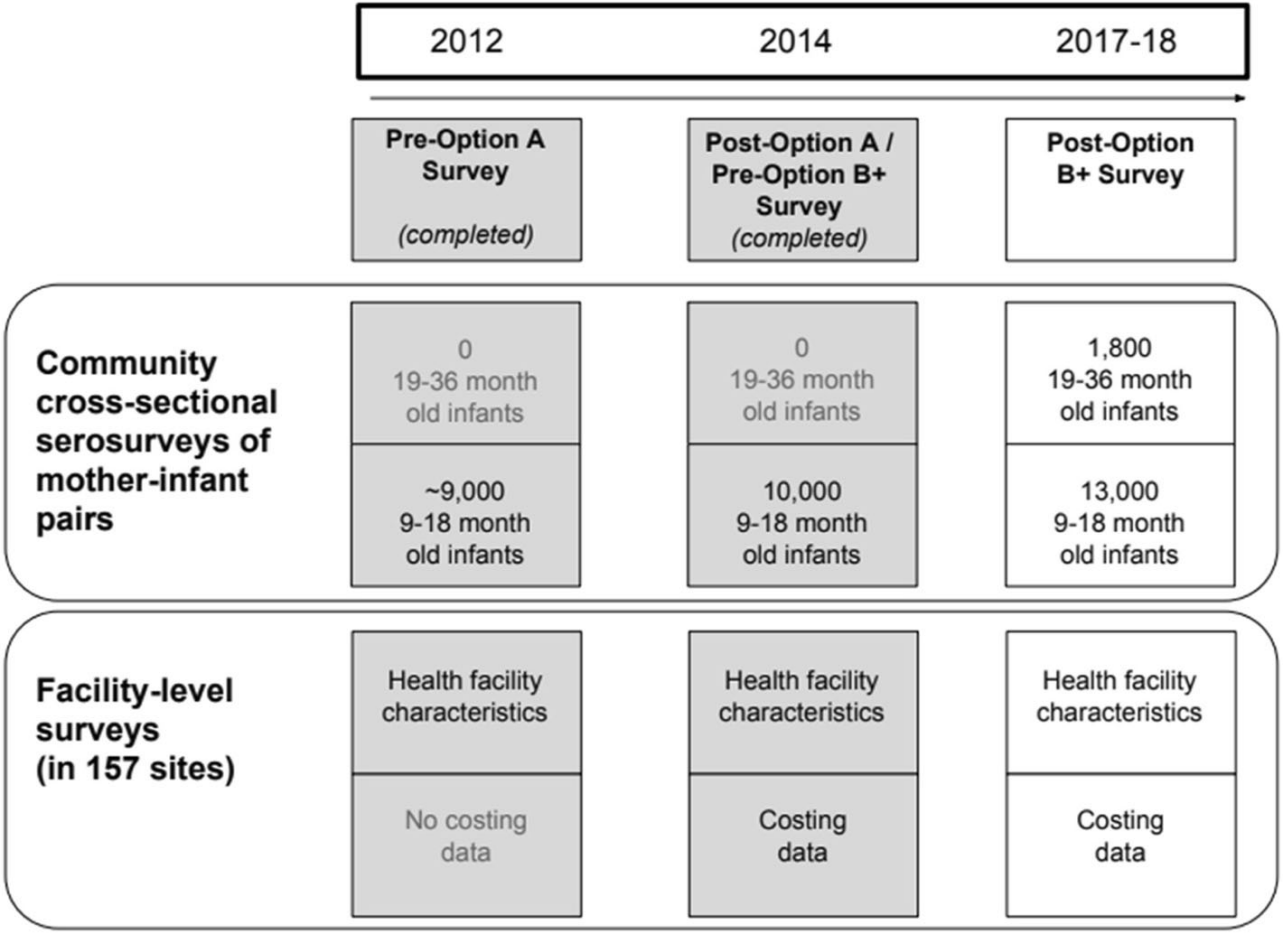
Study Diagram

A timeline of data collection activities for the three cross-sectional serosurveys is summarized in Fig. 1. The study population consists of infants born 9–18 months prior to the surveys (alive or deceased) and their mothers or caregivers (at least 16 years old) living in the catchment areas of 157 randomly selected health facilities in five of Zimbabwe’s ten provinces. The five provinces (Harare, Mashonaland West, Mashonaland Central, Manicaland, and Matabeleland South) were selected in 2012 to include three of the four largest cities in Zimbabwe, rural communities with high and low HIV prevalence, and both major ethnic groups in Zimbabwe (i.e. Shona, Ndebele). The 2017–18 survey will include an additional sample of mothers at least 16 years old who have infants 19–36 months of age in order to examine retention in care. Caregiver-infant pairs are enrolled even if the mother of an eligible child has died or has left the baby for a prolonged period of time (e.g., working elsewhere).

For facility-level surveys, the study population consists of healthcare staff at health facilities in the sampled communities in order to assess facility-level characteristics associated with MTCT (i.e. staff training, infrastructure), and the cost of delivering PMTCT services.

### Sampling strategy

In the first stage of our multi-stage sampling process, we selected catchment areas of health facilities offering PMTCT services within each district of five provinces of Zimbabwe as primary sampling units (PSUs), 157 of 699 catchment areas were randomly selected with probability proportionate to the number of facilities in each district. In 2012 and 2014, the second stage of sampling selected eligible mother-infant pairs living in the catchment areas of these 157 facilities. Women are considered eligible if they are at least 16 years old, able to respond to questions in English or Shona/Ndebele and are living in a catchment area household.

Eligible mother-infant pairs were selected using a pre-determined sampling fraction that was a function of the number of mother-infant pairs identified. In catchment areas with fewer eligible pairs, a greater proportion were sampled to ensure a minimum sample of 50 mother-infant pairs from each catchment area. During the first two survey rounds, eligible mother-infant pairs were identified based on information pooled from: 1) village health workers (VHWs) and 2) immunization registers from selected facilities and neighboring facilities (to identify women residing in sampled facilities who accessed services at adjacent facilities) [9]. Further, mothers identified using (1) and (2) were asked to identify other eligible infants in their neighborhood [9].

For the final survey round in 2017–2018 the investigators chose to adjust the second stage sampling. By this time, a cost-effective strategy engaging community health workers to conduct geographic mapping and to comprehensively identify eligible study participants had been demonstrated by other recent projects in Zimbabwe [19]. Although our previous three-pronged approach, pooling information from VHWs, immunization registers, and respondent driven sampling in each neighborhood, allowed efficient identification of eligible mother-infant pairs without screening all households in a selected CA, it may have systematically excluded eligible mother-infant pairs less likely to access health services. Hence, in discussions with the MOH, we chose to improve the rigor of our sampling procedures, i.e. enumerating catchment area populations by VHWs *prior* to data collection in order to permit more rigorous identification of all eligible study participants. In doing so, we also increase the utility of our findings for MOH efforts aimed at quantifying Zimbabwe’s progress toward elimination of MTCT.

In 2017–18, the second stage of sampling was adapted as follows. Rather than sampling mother infant pairs, we are sampling geographic areas demarcated by the Zimbabwe National Statistics Agency (ZIMSTAT) for use in the national census within each clinic catchment area. These units are referred to as enumeration areas or EAs, and each unit is expected to contain roughly 100 households.

Sampling fractions for EAs within each catchment area were chosen to target selection of roughly 50 mother-infant pairs per catchment area. Sampling fractions used in in the 2014 survey round to select eligible mother-infant pairs in each catchment area were used as a base to select a comparable proportion of EAs from each catchment area for the 2017–18 round. Adjustments were made to sampling fractions to mandate a minimum selection of 7 EAs per clinic catchment area, or all EAs if there were fewer than 7 EAs associated with a clinic catchment area. In each selected EA, village health workers (VHW) are trained by and work in conjunction with survey staff to conduct a full census of all households in the selected EAs. The VHWs and survey teams use area maps to visit all identified structures, and record the number of potentially eligible mothers and infants by visiting households and conducting a short screening survey to ascertain the presence of eligible mother/caregiver-infant pairs. Within the selected EAs, all eligible mothers who have given birth 9–18 months prior, and all infants 9–18 months old are selected for inclusion in the survey. We will also sample 10% of mothers 19–36 months postpartum in order to assess longer term engagement in care following the end of the breastfeeding period.

## Data sources

### Community mapping for the endline survey

At the endline survey, prior to conducting the community census, the survey staff visited each catchment area to collect coordinates of the catchment area boundaries, as these had not been previously captured. The Zimbabwe Census office identified which EAs lay within each catchment area boundary. EAs are selected for inclusion in the survey using the sampling fraction outlined above. In the EAs selected for inclusion, all households are visited and the following screening information is collected from all mothers/caregivers within each household: name, birthdate, whether they are pregnant, if they have any children born in the past 3 years, if they have any children born in the past 3 years that were stillborn or are no longer alive. If mothers/caregivers have a child born in the past 3 years (alive or deceased), the name and birthdate of each child are recorded. Staff will utilize GPS coordinates collected from all households to revisit eligible households at designated dates and times to inform participants that they are selected and therefore invited to participate in the study.

### Endline survey of mothers/caregivers and their babies

Participating mothers/caregivers in both the 9–18 months and 19–36 month postpartum samples are asked to answer an interviewer-administered questionnaire, which captures the mother’s demographic characteristics, healthcare utilization and engagement (including changes in care location), and her experience with antenatal care, HIV testing, delivery, infant feeding, ART and PMTCT prophylaxis for the eligible child. Mothers of 19–36 month old infants who report being HIV-positive will also be asked more detailed information about engagement in HIV care after their pregnancy, their ART regimen, including reasons for any ART interruptions. Living biological mothers 9–36 months postpartum and infants 9–18 months old will be asked to provide dried blood spot samples for HIV testing. Blood spots will be air-dried onto filter papers and stored at room temperature until they are transported biweekly to the National Microbiology Reference Laboratory in Harare. Maternal samples will be tested for HIV-1 antibody using AniLabsytems EIA kit (AniLabsystems Ltd., OyToilette 3, FIN-01720) with all positive specimens confirmed using Enzygnost Anti-HIV 1/2 Plus ELISA and discrepant results resolved by Western Blot. Among HIV-infected mothers, dried blood spot samples will be tested for HIV viral load using Biomerieux Easy Mag/Easy Q platform (Biomerieux, France).

Infant samples will be tested if the infant was born to an HIV-positive mother or if the infant was born to a mother whose sample is unavailable. Infant samples will be tested for HIV with DNA polymerase chain reaction (Roche Amplicor HIV-1 DNA Test 1.5). Mothers who participate in the study will have the option to pick up their test results from their local clinic, and will receive appropriate pre- and post- test counseling for her and her infant’s HIV test results from nurses at each health facility.

### Facility survey

In addition to the community-survey of mother infant pairs, surveyors will administer a questionnaire to the head nurse at each of the 157 health facilities in the selected catchment areas to assess the nature and integration of PMTCT and ART services offered at each facility and to collect information on other health facility characteristics. The survey also includes comprehensive resource utilization and cost information including: 1) the number and type of staff working in PMTCT, 2) PMTCT-related supplies used, 3) the monthly number of ANC and PMTCT clients in the previous calendar year, 4) the amount of staff time allocated to PMTCT services, and 5) the management practices implemented at the facility, such as supervision incentives. Facility records, such as staff rosters, supply stock records, and patient records will be used to corroborate self-reported information when available.

### Study measures

The study will estimate two primary outcomes among 9–18 month mother-infant pairs:

1. HIV-free infant survival: The proportion of infants born to HIV-infected mothers who were alive and HIV-uninfected at 9–18 months of age. The denominator (number of HIV-infected mothers) will be assessed based on either: i) laboratory-confirmed HIV test results, ii) verbal autopsy data, or iii) information recorded on maternal health cards (for deceased or unavailable mothers). To classify deaths as due to AIDS from verbal autopsy data, we will use an algorithm validated in Zimbabwe [20]. The numerator (number of living HIV-uninfected infants) will be assessed based on: i) laboratory-confirmed HIV test results, ii) information recorded on infant health cards, and iii) reports of infants’ deaths.
2. Mother-to-child transmission of HIV (MTCT): The proportion of infants born to HIV-infected mothers who are HIV-infected at 9–18 months of age. The denominator (number of HIV-infected mothers) will be assessed as outlined above. The numerator (number of infants HIV-infected or deceased related to HIV/AIDS) will be assessed based on: i) laboratory-confirmed HIV test results, ii) verbal autopsy data (for deceased infants), and iii) information recorded on infant health cards. A Zimbabwean pediatrician will examine the infant verbal autopsy data and rate the likelihood of each infant death being HIV-related (on a 5-point scale ranging between ‘very unlikely’ and ‘very likely’) based on: gestational age at delivery, birth weight, infant age at death, symptoms indicative of common opportunistic infections in children, along with the chronicity of their illness. She will take account of factors that affect likelihood of MTCT (e.g. breastfeeding). ‘Likely’ and ‘very likely’ cases were classified as infant HIV/AIDS-related deaths.

Secondary outcomes:

3) Viral suppression: The proportion of HIV-infected mothers who are virally suppressed 9–18 months postpartum. The denominator (number of HIV-infected mothers) will be assessed as outlined above. The numerator will be assessed based on laboratory viral load test results. Mothers with viral load test results indicating < 1000 copies/ml will be classified as virally suppressed.
4) Postpartum engagement in care (among mothers 19–36 months postpartum): The proportion of HIV-infected mothers who were initiated on ART and who continued ART. The denominator (number of HIV-infected mothers who were initiated on ART) will be assessed using: i) laboratory-confirmed HIV test results, or ii) information recorded on maternal health cards, when available, and iii) self-reported ART status. The numerator (number of HIV-infected mothers who were initiated on ART and continued ART after 19–36 months postpartum) will be assessed based on self-reported ART status at the time of the survey. Additionally, we will calculate the average time between delivery and discontinuation of ART among those mothers who did not continue ART.
5) Heterogeneity of the impact of Option B+ on HIV-free survival and MTCT by the extent of integration of PMTCT and ART services at health facilities: Impact heterogeneity of Option B+ will be assessed using community-level cross-sectional serosurvey data as well as data on health facility characteristics from the 157 catchment areas selected as PSUs. The extent of integration of PMTCT and ART services at the facility level will be measured using multiple dichotomous variables such as: whether the facility offers both antenatal and labor/delivery services, whether the facility can initiate pregnant women on ART, whether the facility can continue women on ART after a certain number of months after delivery, whether the facility can offer ART services to male partners, and whether the facility can offer ART services to infants and children. Principal component analysis will be employed to estimate the factor loadings of these variables, in order to calculate an overall integration score per facility.
6) Incremental cost effectiveness of Option B+: Assessed using effectiveness data (from the community-level cross-sectional serosurveys) along with costing data (from the 157 health facilities surveys). We will estimate the incremental cost-effectiveness ratio (ICER) of option B+ with respect to option A, and the ICER of option B+ with respect to pre-option A guidelines, as the ratio of the difference in costs over the difference in annually-adjusted effectiveness (for each of the two main outcomes: HIV-free infant survival and MTCT).

### Analyses

All statistical analyses will be conducted using the software package STATA version 15 (StataCorp, Texas). The data will be weighted to account for the multi-stage stratified cluster design and survey non-response. For the first two rounds of the survey weighting was done to account for the selection of catchment areas within districts, mother infant pairs within catchment areas and for survey non-response. For the final round survey, weighting is the same for the selection of clinics within districts, but differs for the later stage weighting. For this survey, weights will be constructed for the probability of being in a selected EA within the catchment area, the probability of complete census data within EAs (census non-response), and survey non-response among identified eligible mother infant pairs. GPS coordinates collected for each structure in selected EAs and will be used to assess whether census non-response is associated with distance from the clinic or distance from the center of the EA.

To assess the population-level impact of Option B+ (Objective 1), we will estimate and compare the population-level differences in MTCT and HIV-free infant survival among infants born after Option B+ is implemented (2017–18 estimates) and those born under the standard of care prior to Option A estimates (2012 estimates). Next, we will estimate the differences in these outcomes between infants born after Option B+ is implemented (2017–18 estimates) and those born under Option A guidelines (2014 estimates). These estimates will be generated by treating the two conditions compared in each analysis as matched pair observations for each catchment area, adjusting only for the sampling design through weighting. As with all pre-post evaluation designs, there is a concern that temporal trends, unrelated to the intervention, may be responsible for observed differences in the outcomes. To address this concern, we will also estimate differences in MTCT and HIV-free survival proportions adjusted for facility- and individual-level covariates not affected by the intervention. For example, in addition to demographic and socioeconomic characteristics such as the age of the mothers and infants, we will adjust for women’s ART use before pregnancy. To adjust for these factors, we will apply targeted maximum likelihood estimation, as well as standard parametric regression and inverse probability weighted methods.

To assess heterogeneity of the impact of Option B+ on HIV-free survival and MTCT among infants 9–18 months of age by the extent of integration of PMTCT and ART services at health facilities (Objective 2) we will first describe the variability in service integration, and characteristics of the facilities and catchment populations. We will capitalize on the variation of the integration score and other facility characteristics across the catchment areas to examine whether they affect the effectiveness of Option B+. We will describe differences in HIV-free survival by these facility-level characteristics. We will employ two identification strategies for these analyses. First, we will model 2017–18 outcomes adjusted for 2012 characteristics in each facility using ordinary least squares regression with HIV-free infant survival (or MTCT) as the aggregate continuous outcome. Similarly, we will model 2017–18 outcomes adjusted for 2014 characteristics. Second, we will apply a “difference-in-difference” approach, in which the outcomes will be defined as a) changes in HIV-free survival (or MTCT) between 2017 and 18 and 2012 in each catchment area, as well as b) changes in the same outcomes between 2017 and 18 and 2014.

Analysis for the retention of mothers in ART services after 19–36 months postpartum (Objective 3) will be primarily descriptive due to small sample size (e.g. 10% of eligible mothers 19–36 months postpartum). We will explore possible variations in the proportion of mothers who continued ART after weaning by whether the HIV-infected mother was or not on ART prior to PMTCT, to assess whether asymptomatic mothers were retained in care. We will also examine the average time between weaning and discontinuation of ART among those mothers who did not continue ART.

To determine the cost-effectiveness of the PMTCT program implementing Option B+ guidelines (Objective 4), we will estimate an incremental cost-effectiveness ratio (ICER). The ICER will be a ratio of the incremental cost of Option B+ over the incremental impact of a) the standard of care before Option A, and b) Option A. The costs will be expressed in monetary terms, and the benefits will be expressed in terms of HIV-free infant survival and MTCT rate. Detailed retrospective input and output data, as well as prices information will be collected from facilities We will estimate the facility-level annual economic costs of producing each modality of PMTCT services—prior A, A, B + —by adding the annual cost, i.e. monthly quantities multiplied by their prices, of three essential input categories used under each modality: personnel, ARV drugs and HIV tests kits. We will also estimate the average costs per woman enrolled in the PMTCT program, per facility, under each option. In 2012 and 2014 we collected data for standard of care pre-Option A and Option A, and in 2017–18 we will collect data for Option B+, for 1 year of implementation of each alternative retrospectively. Input data will include staff time devoted to the PMTCT programs, consumables such as HIV test kits and ART drugs. Unit prices and salaries for all these inputs will also be collected. Total costs for the different PMTCT models will then be estimated. For the same periods for which input data is collected, we will also gather data on the number women on each PMTCT alternative program, and will estimate the average cost per woman enrolled in PMTCT, per facility.

### Statistical power

We hypothesized that differences in HIV-free infant survival and MTCT between Option B+ (2017 estimates) and the pre-Option A standard of care (2012 estimates) will be greater than the differences between Option B+ (2017–18 estimates) and Option A (2014 estimates). Therefore, sample size calculations for the community surveys were conducted targeting the comparison between 2014 and 2017–18 estimates, as described below.

Sample size calculations were based on the formula for unadjusted comparison of proportions within matched pairs (where a “pair” is two measurements of the outcome within the same catchment area e.g. MTCT in a given facility in 2014 and in 2017–18) [21]. Findings from the 2012 survey estimate an average ANC prevalence of 12.5% among mothers in our catchment areas, with 9% of HIV exposed 9–18 month old infants being infected and 10% of HIV-exposed infants being deceased or infected with HIV [9]. In 2014 ANC prevalence was 13.6% among mothers in our catchment areas and the proportion of HIV-exposed infants who were deceased or HIV-infected had dropped to 4.8% [22]. In Zimbabwe the national targets for Option B+ are 95% HIV-free infant survival and less than 5% MTCT. Consequently, we calculated the sample size needed to detect a reduction from 2014 outcome proportions to 3.4% HIV infection or death in 2017–18.

Based on our 2012 and 2014 survey experience, we presume the 2017–18 survey will have a survey refusal rate of 2% and a value of 0.25 for the coefficient of variation (k_m_). Using a two sided-test at a 5% level of significance and a desired power of 80%, we would require a harmonic mean greater than 4 HIV-exposed infants (i.e., infants whose mothers are HIV-infected) per catchment area. We expect that the prevailing trend of decreasing ANC prevalence will continue [23] and therefore assume that ANC prevalence will have decreased to 11.5% by 2017–18. Taking into account variability in both catchment area size and underlying HIV prevalence among pregnant women across PSUs (again, based on our 2012 and 2014 data) we estimate that we will need a sample 13,000 pairs in 2017–18. Using the above assumptions we would have 80% power to detect a difference in HIV-free infant survival between Option A and Option B+.

For mothers at 19–36 months post partum, under the assumption of a 11.5% HIV prevalence among mothers in 2017–18, and taking into account variation in prevalence and refusal rates similar to those observed in the 2012 and 2014 surveys, we estimated that, in order to measure the proportion of mothers who continued ART after weaning, we would require a sample of approximately 1800 mother-infant pairs, of which approximately 200 mothers are expected to be HIV-infected.

## Discussion

Despite the current global goal to eliminate pediatric HIV, to date few countries have assessed the population level impact of their PMTCT programs. We planned an ambitious study to measure the real-world effectiveness of Zimbabwe’s National PMTCT programme using innovative methodology to estimate the impact of PMTCT programs Option A and B+. Our design addresses key disadvantages of facility based evaluations of PMTCT programs by allowing us to assess HIV-free infant survival and MTCT at 9–18 months, thus taking into account HIV transmissions occurring during pregnancy, labor and breastfeeding among both women who access PMTCT services and those that do not [17].

Methodological issues in existing PMTCT effectiveness estimates limit our ability to assess progress towards global and country-level PMTCT elimination goals. WHO impact assessment guidelines suggest measurement of PMTCT program impact using mathematical/dynamic modeling, analysis of program data, serial cross-sectional serosurveys at 6–8 week immunization visits, cohort studies of mother-infant pairs attending health facilities, or serial cross-sectional serosurveys among community samples of infants [24]. In addition, WHO recommendations identify the MTCT rate and the HIV-free infant survival as key outcomes to measure effectiveness, with the latter being the gold-standard [25], as it accounts for infant infections *and* deaths. Outcomes should be measured post-breastfeeding, ideally at 18 months [15], as 15–20% of MTCT occurs during breastfeeding [3]. Despite these recommendations, most PMTCT effectiveness studies have used routinely collected facility-level program data and thus have not assessed population level impact [26]. Facility-based studies use existing health system mechanisms to identify infants, reducing the burden of capturing a study sample, which likely explains their predominance in evaluations. While these studies provide valuable information about the programs’ outcomes among their users, they do not measure population-level effectiveness. If coverage of such services (e.g., antenatal care, delivery, postnatal care) increases across developing countries, existing data sources may become suitable for PMTCT impact assessments. Meanwhile, however, if women not accessing services are more likely to be HIV-infected than women using health services, even if service uptake is ≥80%, facility-based studies could still overestimate program impact on MTCT.

Existing studies measuring MTCT do not capture infant deaths and hence likely overestimate impact (e.g., given high mortality among HIV-exposed and HIV-infected infants) [3, 27] and/or mask the intervention’s unintended effects (e.g., increase of infant deaths due to replacement feeding) [28, 29]. Studies utilizing prospective cohorts of mother-infant pairs allow assessment of MTCT and HIV-free infant survival, but is sensitive to LTFU, which reduces validity, and cohort effects where study participants may have better outcomes due to the care and follow-up they receive, or as a result of being observed (i.e., Hawthorne effect) [14]. Furthermore, the high cost of prospective follow-up remains a financial barrier for many impact evaluations.

We address these methodological gaps by conducting an impact evaluation of Option B+ using a community-based design that does not rely on facility level data, thus capturing PMTCT only among women who receive care at health facilities. While our serial community-based cross-sectional design has been used to assess Nevirapine-based PMTCT programs in four African countries [30], Option A in Zimbabwe [31], and Option B in Rwanda [32] and Zambia [28] it has not been used to assess the effectiveness of Option B+.

In addition to the lack of evidence on community effectiveness, no studies have assessed whether there is heterogeneity, based on type pf service delivery, in the potential impact of Option B+. The population-level effectiveness of PMTCT programs implementing Option B+ is likely to vary between health facilities [15] For example, providing lifelong ART to all women with HIV who enter the PMTCT cascade of services, as required by Option B+, requires decentralization of ART services as well as integration of PMTCT and ART services. The level of integration of these services may affect the program’s impact, as linkage to and retention in treatment and care is likely to be lower in facilities that refer women to ART centers compared to facilities are able to initiate women on ART. We know of no previous studies that have been able to determine impact heterogeneity of Option B+; specifically, we will conduct a novel analysis combining health facility data with aggregate population-level MTCT data from facility catchment areas. This analysis will also inform policymakers about which facility characteristics are associated with higher rates of HIV-free infant survival and less MTCT at 9–18 months.

Finally, the population-level benefits of Option B+ are predicated on mothers being retained in care for life, and our design will be one of the first population-based assessment of retention in HIV care up to 3 years postpartum among mothers who received Option B+. By simplifying service delivery and reducing the number of steps that mothers need to negotiate, Option B+ is expected to improve retention in care and adherence to ART. Given that the postulated benefits of Option B+ are predicated on HIV-infected mothers staying on ART beyond breastfeeding, our study design will test this assumption.

Our study is subject to limitations. Although our estimates account for transmissions occurring during the first 9–18 months of breastfeeding, HIV-exposed infants may still be breastfeeding at the time of the survey (median duration of breastfeeding is 17.8 months in Zimbabwe) [33]. Estimates of HIV-free survival for 9–18 month old infants may therefore overestimate HIV-free survival at 24 months (at the end of breastfeeding) and may result in an underestimation of MTCT. Nonetheless, estimates restricted to all HIV-exposed infants no longer breastfeeding at the time of our 2012 survey were comparable: 91.9% (95% CI: 86.8–95.1) HIV-free infant survival and 8.1% (95% CI: 4.9–13.2) MTCT [9].

If there is inadequate coverage of the full census, or inconsistency in the ability of health workers and survey staff to survey eligible mother infant pairs, the sample in our endline survey might represent a biased population. Recruitment in the community could vary during the periods of the year when there is greater temporary migration related to holidays or agricultural activities (going to the rural home for planting or harvest). In addition, although sampling procedures were modified in 2017–2018 to increase the rigor of our methodology, these changes may impact the comparability of our findings to previous survey rounds. Despite this, the added value of a more comprehensive strategy for identifying our target population of mother-infant pairs, combined with the utility of our modified approach in supporting MOH efforts to quantify Zimbabwe’s progress toward elimination, warrant this change. Finally, data will only be collected in five of ten provinces, although these were widely dispersed across the country and included major cities. Nevertheless, our findings from the 2012 survey were consistent with national-level data from the 2010–2011 ZHDS that similarly estimated that 12% of pregnant women were HIV-infected [33]. Despite these limitations, our unique design utilizing cross-sectional serosurveys at three time points will be one of the first impact evaluations of Option B+ using a community-based design. Our results will have direct relevance for Zimbabwe and other developing countries, as they will provide essential data to better understand the population-level impact and cost effectiveness of Option B+ and assess progress towards global MTCT elimination goals.
